# A Novel MRI-Based Risk Stratification Algorithm for Predicting Postoperative Recurrence of Meningioma: More Benefits to Patients

**DOI:** 10.3389/fonc.2021.737520

**Published:** 2021-10-19

**Authors:** Rufei Zhang, Xiaodan Chen, Jialing Cai, Peirong Jiang, Yilin Chen, Bin Sun, Yang Song, Lin Lin, Yunjing Xue

**Affiliations:** ^1^ Department of Radiology, Fujian Medical University Union Hospital, Fuzhou, China; ^2^ Department of Radiology, Fujian Medical University Cancer Hospital, Fuzhou, China; ^3^ School of Medical Technology and Engineering, Fujian Medical University, Fuzhou, China; ^4^ MR Scientific Marketing, Siemens, Healthineers Ltd, Shanghai, China

**Keywords:** meningioma, recurrence, prediction model, relative apparent diffusion coefficient, heterogeneous tumor enhancement, pathological grade

## Abstract

Pathological grading of meningioma is insufficient to predict recurrence after resection and to guide individualized treatment strategies. One hundred and thirty-three patients with meningiomas who underwent total resection were enrolled in this retrospective study. Univariate analyses were conducted to evaluate the association between factors and recurrence. Least absolute shrinkage and selection operator (Lasso) was used to further select variables to build a logistic model. The predictive efficiency of the model and WHO grade was compared by using receiver operating characteristic curve (ROC), decision curve analysis (DCA), and net reclassification improvement (NRI). Patients were given a new risk layer based on a nomogram. The recurrence of meningioma in different groups was observed through the Kaplan-Meier curve. Univariate analysis demonstrated that 11 risk factors were associated with prognosis (P < 0.05). The result of ROC proved that the quantified risk-scoring system (AUC = 0.853) had a higher benefit than pathological grade (AUC = 0.689, P = 0.011). The incidence of recurrence of the high risk cohort (69%) was significantly higher than that of the low risk cohort (9%) by Kaplan-Meier analysis (P < 0.001). And all patients who did not relapse in the high risk group received adjuvant radiotherapy. The novel risk stratification algorithm has a significant value for the recurrence of meningioma and can help in optimizing the individualized design of clinical therapy.

## Introduction

Meningiomas are the most common primary intracranial tumors, accounting for 38.3% of all tumors of the central nervous system, and are continuing to increase in incidence with the aged tendency of the population (1). They result in severe neurological morbidity by having a space occupying effect on adjacent brain regions. Complete surgical resection of the meningioma is the first choice of treatment (2).

Up to now, the most reliable clinical factor for recurrence of meningioma after radical surgical resection is the World Health Organization (WHO) grade of the tumor (2–4). The WHO grade is currently used to guide the development of strategies for postoperative radiotherapy and follow-up (2, 4). However, despite radical surgical resection, some benign meningiomas (WHO grade I) have early recurrence after surgery and exhibit aggressive biological behavior, resulting in chronic courses of diseases and treatment-related complications (3). In contrast, some high grade tumors (WHO grade II, III) have indolent tumor behaviors, which suggests that histopathological grading alone is not enough to achieve optimal risk stratification. Therefore, it is clinically difficult to predict postoperative recurrence and guide individual decisions only by tumor classification and it is particularly important to clarify the risk changes within the inter-layer variability (5–8). Although numerous studies have identified the histological, clinical, and radiological parameters regarding aggressive meningioma behavior, the accurate prediction of postoperative recurrence of meningioma remains challenging and requires further research (4–6, 8, 9).

The purpose of our study was to generate and validate an MRI-based prediction model of meningioma recurrence after surgery that could be coupled with potential clinical prognostic factors and histopathological grade. We hypothesis that the new prognostic stratification constructed by this new model might individualize decisions regarding the need for postoperative therapeutic interventions and support the optimization of treatment strategies for patients.

## Materials and Methods

### Patient Characteristics

Patients with meningioma who underwent surgical resection at the department of neurosurgery of the Fujian Medical University Union Hospital between June 2010 and December 2020 were enrolled in this retrospective study. This study was approved by the local ethics committee of the hospital, and the requirement of written informed consent was waived. The inclusion criteria for this study were patients who underwent preoperative and postoperative craniocerebral MRI examination and were pathologically confirmed as meningioma in our hospital. We excluded patients with prior craniocerebral radiation therapy, neurofibromatosis type 2 (NF2), preoperative imaging outside our hospital, poor image quality, or less than 2 years of clinical and radiological follow-up in our hospital.

### Imaging Acquisition

MR images were performed on 1.5T (Siemens, MAGNETOM Amira, n = 67) or a 3.0T (GE Healthcare, Discovery MR750, n = 57, or Siemens, MAGNETOM Trio, n = 9) MR scanners according to a standard institutional protocol in our hospital. MRI examinations included axial T1-weighted spin-echo, T2-weighted, T2 fluid attenuated inversion recovery (FLAIR), T2-weighted gradient-recalled echo, diffusion weighted images (DWI), and apparent diffusion coefficient (ADC), T1-weighted images after contrast administration. Detailed MRI acquisitions were provided in **Supplementary Appendix E1**.

### Pathological Examination

The detailed histopathology reports and the histopathologic slides of surgical specimens were re-evaluated by a neuropathologist (with more than 10 years’ experience), and the pathologic diagnoses and tumor grading were based on the 2016 WHO Classification of Tumors of the Central Nervous System (10). According to 2016 WHO criteria, meningiomas can be classified into three grades based on histological and cytological characteristics: WHO grade I with < 4 mitoses/10 high power field (HPF), WHO grade II with 4–19 mitoses/10 HPF and/or brain invasion, and WHO grade III with ≥ 20 mitoses/10 HPF (**Supplementary Table**). For every case, the representative hematoxylin-eosin (HE) staining slide which was most representative of the mitotic count or other grading standards was determined and calculated for the pathological grade by the experienced neuropathologist. Subsequently, WHO grade I meningiomas was defined as low grade meningiomas, and WHO grade II and III meningioma were defined as high grade meningiomas based on previous literature (3, 6, 11, 12).

### Recorded Variables

All clinical data and imaging features were evaluated by two neuroradiologists (with more than 10 years’ experience) who were not informed of the prognosis. Patients’ gender, age at the time of surgery, pre-operative Karnofsky Performance Scale (KPS) scores, Ki-67 index, and administration of adjuvant radiation were determined from clinical records. The grade of resection was determined by reviewing surgical records, MRI, and best clinical judgment in the absence of explicit instructions.

T2 hyperintensity was determined by comparison with the gray matter signal. Tumor sites were divided into “skull base” and “non–skull base” lesions (including convexity and falcine/parasagittal meningiomas and tumors arising from other intracranial non–skull base locations). The maximum diameters were measured in 3 directions (axial, coronal, and sagittal) on MRI, and the tumor and edema volume were calculated according to the elliptical sphere volume formula. The edema index was defined as the ratio of the volume of edema to the sum of the tumor plus edema, EI = (V_Edema_+V_Tumor_)/V_Tumor_). Regular tumor shapes included round or oval shapes, other shapes were irregular. Tumor calcification was diagnosed by reference to preoperative CT images. Heterogeneous tumor enhancement did not include heterogeneity due to cystic change or calcifications. The presence of the arachnoid layer was examined with T2 predominant MR screenings. Observations of dural tail sign and cystic change were made using T1-weighted contrast images. Venous sinus invasion and bone change were determined by combining radiological data and surgical records.

For ADC measurement, the region of interest (ROI) was placed at the largest level of the tumor while avoiding calcification and cystic change and varying with the tumor size (20 – 600 mm^2^). To normalize individual variance, the ADC value of the tumor was divided by the corresponding value of contralateral semioval center normal appearing white matter (NAWM) to calculate the relative ADC (rADC) (13).

As the current gold standard, biopsy is used to determine tumor recurrence, but it might lead to more stress and pain for the patient. Fortunately, the recurrence can also be determined by other non-invasive methods, such as follow-up enhanced MRI scans. Follow-up MR images showing new enhanced lesions appeared in the surgical area can be considered as evidence of tumor recurrence (**Supplementary Figure**). In previous studies, follow-up enhanced MR has been used as one of the diagnostic criteria for determining tumor recurrence (3, 11–13). Accordingly, tumor recurrence was determined by at least one of the following conditions in this study: (I) follow-up enhanced MRI scans indicated tumor recurrence; (II) tumor recurrence was pathologically confirmed by reoperation or biopsy.

### Statistical Analyses

All statistical analyses, model generation, and model validation were carried out using statistical software SPSS (version 23.0) and R (version 4.0.3). The interobserver reliability of categorical data was determined by the Cohen kappa (κ) coefficient, whereas the consistency of consecutive data was determined by the intra-group correlation coefficient (Cohen κ values ranged from 0.757 to 0.931 and ICC from 0.831 to 0.983).

The distribution uniformity of the continuous parameters was evaluated by the Kolmogorov-Smirnov test. The mean ± standard deviation was used to represent the normally distributed data, and the median and interquartile range (IQR) was used to express non-normally distributed data. Chi-square test (or Fisher exact test) and Mann–Whitney U test (or Student’s t-test) were performed for categorical and continuous data respectively. The overfitting problem caused by multicollinearity was solved by the regularization method. Based on univariate analysis, Lasso regression cross-validation was further used to screen out meaningful variables and the logistic regression equation was finally calculated. Then, based on the proposed Lasso-logistic regression model, a nomogram was generated. The collinearity diagnosis was performed by calculating the variance inflation factor (VIF) of the variables in the nomogram. Variables with VIFs > 10 indicated severe multicollinearities. Area under curve was used to measure the discriminability of the nomograms. Calibration curve was plotted through bootstrap (1,000 resamplings) to determine the conformity between the predicted probability and the actual probability. In addition, the nomogram was conducted internally validated by the Bootstrap validation with 1000 resamplings. The clinical utility of the model was measured by decision curve analysis. Delong test was used to measure the differences in ROC curves between the prediction model and the pathological grade. Subsequently, the patients were grouped according to NRI. The relationship between benefits and risks brought by the model was evaluated by DCA. Finally, the recurrence of meningiomas in different groups was observed by Kaplan-Meier analysis. Unless otherwise specified, P value < 0.05 was considered statistically significant.

## Results

### Patient Characteristics

**Figure 1:**
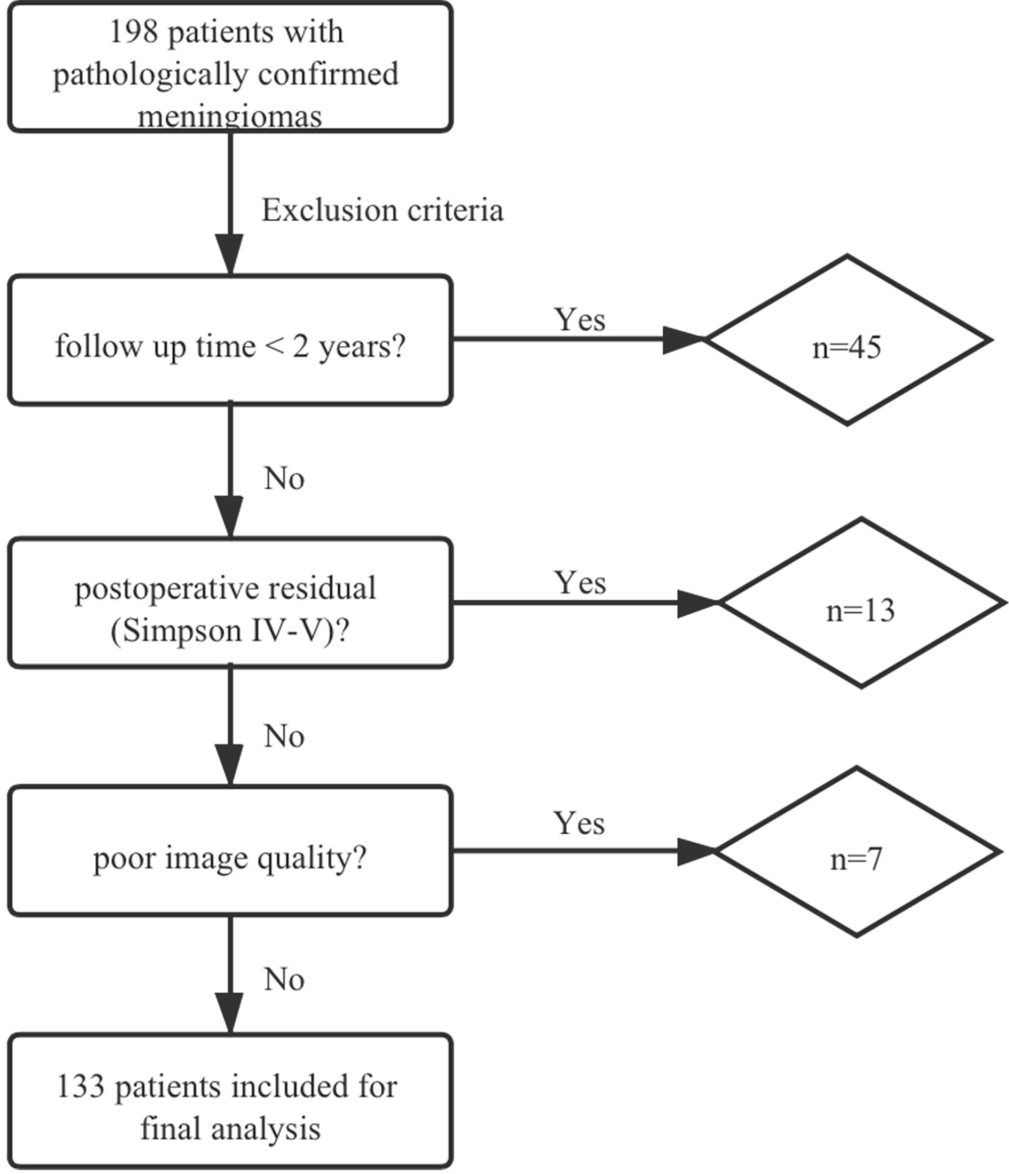
Flowchart of the patient selection process.

In total, 198 patients with meningiomas were diagnosed pathologically in our hospital. Forty and five cases were excluded due to the follow-up time of less than 2 years. Thirteen cases were excluded due to postoperative residual (Simpson IV-V), and 7 cases were excluded because the image quality did not meet the research requirements (2 patients with poor DWI/ADC imaging quality (air-bone interface or motion artifacts) and 5 patients with incomplete MRI) (**Figure 1**).

Finally, 133 patients with a median age of 52 years (range 13–78 years), including 94 females (71%) and 39 males (29%), were enrolled in this study. The median follow-up duration of non-recurrence patients was 40 months (IQR = 31–59 months). Of the 133 patients included in the analysis, the most predominant lesion of 3 patients with multiple lesions was selected for the analysis. The pathological classification included 96 low grade (WHO grade I, n = 96, 72%), 37 high grade (WHO grade II, n = 33, 24%; WHO grade III, n = 4, 3%) meningiomas. Eight (8%) low grade patients and thirteen (35%) high grade patients received adjuvant radiotherapy. Twenty cases (15%) experienced recurrence after radical surgery. **Table 1** summarizes the baseline clinical, radiological, and histopathological data of patients with meningioma.

**Table 1:** Patient characteristics and univariate analysis of the risk of recurrence.

Factor	Recurrence	Non-Recurrence	P
Sex			0.095
Female	11	83	
Male	9	30	
Preoperative SKP			0.280
≥ 70	8	60	
< 70	12	53	
Pathological grade			< 0.001^***^
I	8	88	
II-III	12	25	
Tumor shape			0.017^**^
Regular	3	49	
Irregular	17	64	
Cystic change			0.038^*^
Yes	8	19	
No	12	94	
Heterogeneous tumor enhancement			0.001^**^
Yes	9	14	
No	11	99	
Bone change			0.386
Yes	7	29	
No	13	84	
Dural tail sign			0.405
Yes	14	68	
No	6	45	
Tumor location			0.160
skull base	10	38	
non-skull base	10	75	
Venous sinus invasion			0.023^*^
Yes	9	24	
No	11	89	
T2 hyperintensity			0.953
Yes	8	46	
No	12	67	
Arachnoid layer			0.009^**^
Yes	10	88	
No	10	25	
Calcification			0.499
Yes	1	15	
No	19	98	
Adjuvant radiotherapy			0.119
Yes	6	15	
No	14	98	
Age (years)	59.50 (48.5-67.50)	51 (45.5-58)	0.014^*^
Tumor volume (cm^3^)	87.35 (24.74-133.59)	28.83 (12.83-72.89)	0.005^**^
Peritumoral edema volume (cm^3^)	46.59 (23.70-99.48)	2.55 (0-62.44)	0.004^**^
Peritumoral edema index	1.79 (1.30-2.40)	1.07 (1.00-2.17)	0.016^*^
Relative apparent diffusion coefficient	0.99 ± 0.20	1.19 ± 0.18	< 0.001^***^

^*^P < 0.05, ^***^P < 0.01, ^***^P < 0.001.

### Univariate Analysis

On univariate analysis, pathological grade (I *vs*. II-III, P < 0.001), tumor shape (regular *vs*. irregular, P = 0.017), peritumoral edema volume (P = 0.004), peritumoral edema index (P = 0.016), tumor volume (P = 0.005), heterogeneous tumor enhancement (P < 0.001), cystic change (P = 0.038), venous sinus invasion (P = 0.023), arachnoid layer (P = 0.009), age (P = 0.014), and rADC (P < 0.001) were significantly associated with postoperative recurrence (**Table 1**). There were no significant associations between recurrence and sex, preoperative SKP, bone change, dural tail sign, tumor location, T2 hyperintensity, tumor calcification or adjuvant radiotherapy.

### Feature Selection and Lasso-Logistic Model Construction

**Figure 2:**
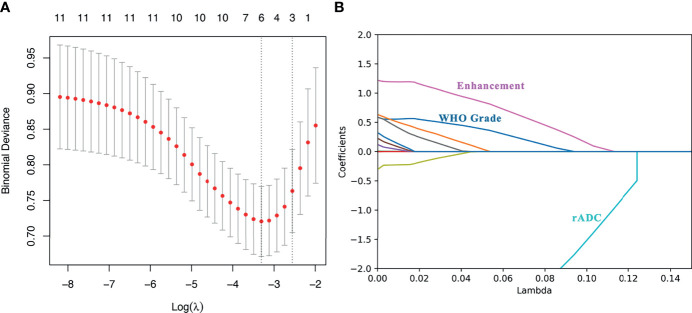
Lasso regression for predicting postoperative recurrence of meningioma. **(A)** The number of independent variables of the model with good performance was 3; **(B)** The three variables were WHO grade, heterogeneous tumor enhancement and rADC value, respectively.

**Figure 3:**
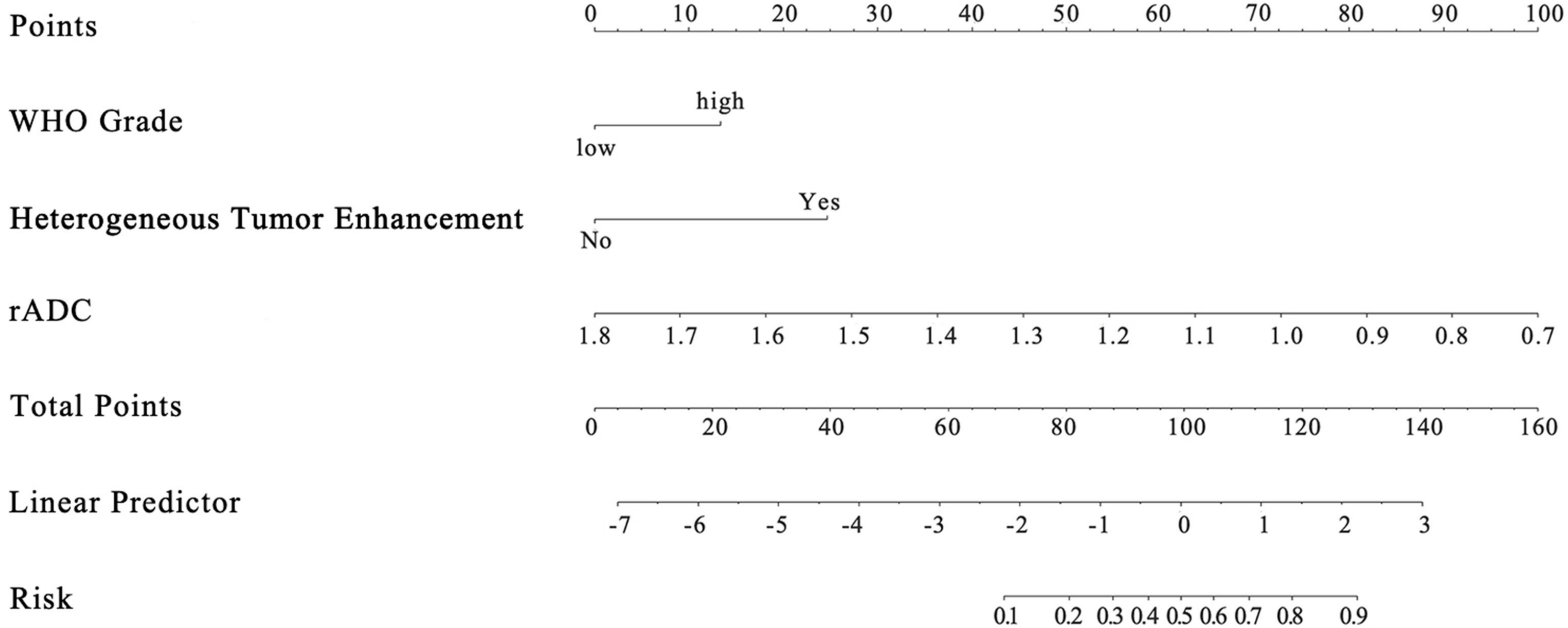
The novel algorithm nomogram for predicting postoperative recurrence of meningioma.

Among the 11 candidate features, potential predictive variables were obtained by dimensionality reduction through Lasso regression cross validation. Variables were selected based on a model with excellent performance and the least number of independent variables given by Least Squares Error (**Figures 2A, B**), and then the Lasso-logistic model was established. The formula for Lasso was provided in **Supplementary Appendix E2**. Pathological grade, heterogeneous tumor enhancement, and rADC were significantly correlated with the actual postoperative status, and these three variables showed significant differences between patients with and without recurrence (P < 0.001). By using the collinearity diagnosis, the VIFs for pathological grade, heterogeneous tumor enhancement, and rADC were less than 10 (WHO grade: 1.0370; Tumor enhancement: 1.0360; rADC: 1.0726), indicating no severe collinearity existing in these factors. These independent predictors were used to construct a nomogram for the prediction of tumor recurrence (**Figure 3**). The formula for the nomogram was computed by:


Nomogram score = 13.33824×WHO Grade level+24.67939×Tumor Enhancement type−90.909090909×rADC value+163.636363636

In the given nomogram score formula, the value of low level (WHO I grade) was 0 and that of high level (WHO II, III grade) was 1. For tumor enhancement type, the value of homogeneous enhancement was 0 and that of heterogeneous enhancement was 1.

### Evaluation and Validation of Lasso-Logistic Model

**Figure 4:**
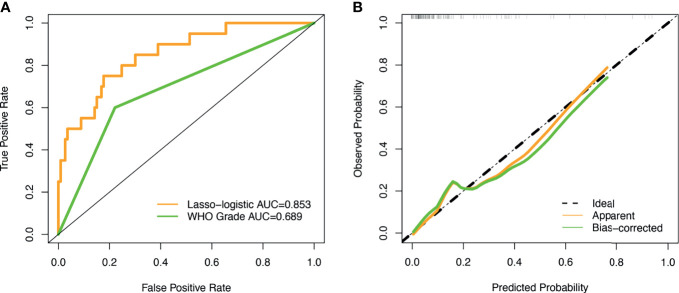
ROC curve and calibration curve. **(A)** The AUC values of the novel algorithm and WHO grade was 0.853 (yellow) and 0.689 (green), respectively; **(B)** The calibration curve demonstrated that consistency between the predicted risk of recurrence probability and the actual probability by the nomogram.

**Figure 5:**
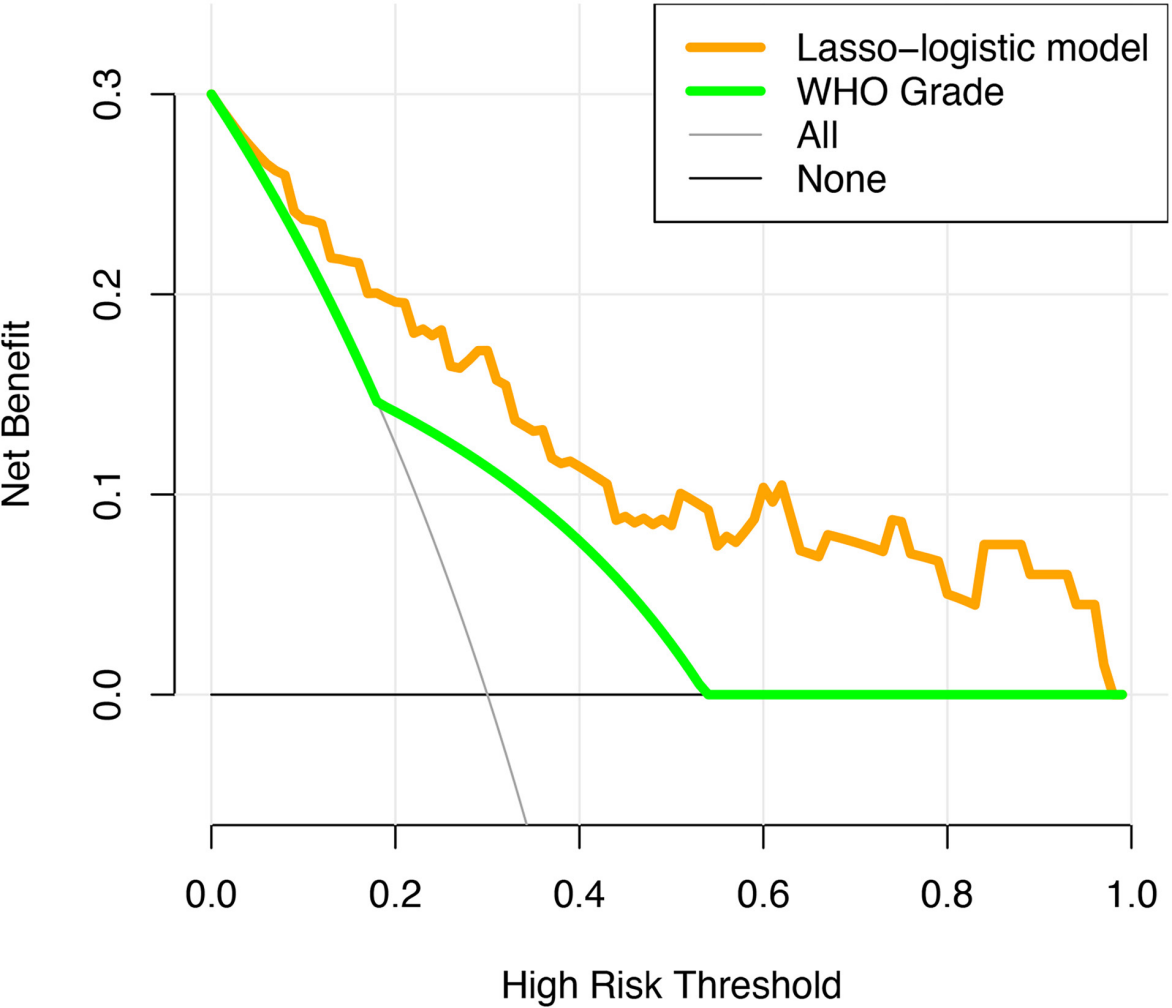
The decision curve analysis for the novel algorithm and WHO grade. The yellow and green dotted lines represent the decision curve of the novel algorithm and WHO grade, respectively.

The unadjusted AUC value based on the prediction model was 0.853 (95%CI: 0.764-0.942). The internal validation of the model was executed by the bootstrap resampling method. The corrected AUC value (AUC = 0.824) was calculated by using the 1000 times bootstrap. Besides, the calibration curve showed that the probability of recurrence predicted by the nomogram was in good agreement with the actual probability (**Figure 4B**). The insignificant statistics (P = 0.439) of H-L test revealed that there was no significant deviation from an ideal fitting. Further, Delong’s test showed that the nomogram had significantly better predictive power than pathological grade alone (P = 0.011). The ROC curve of the model and pathological grade were shown in **Figure 4A**. NRI quantified the extent to which the addition of risk factors (two radiological features) led to an improved classification of risks. The decision curve for the nomogram and the pathological grade alone were used to assess the clinical utilities. **Figure 5** showed that the area under the decision curve of the nomogram (yellow) was higher than that of the pathological grade alone (green).

### Novel Risk Stratification for Recurrence

**Figure 6:**
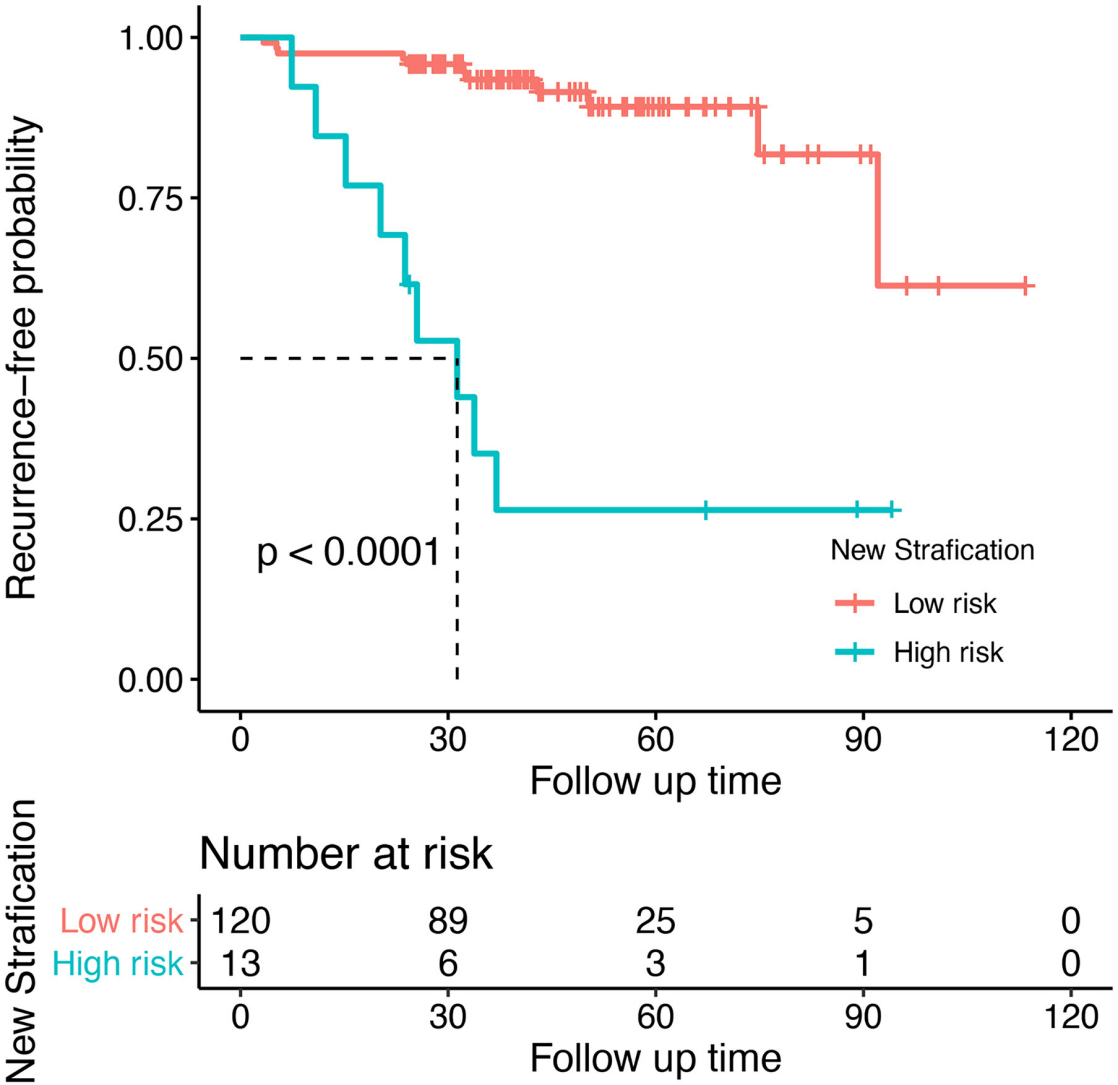
Recurrent risk stratification for patients with meningioma after surgery. Kaplan-Meier curve showed significant differences in postoperative recurrence between the two groups (P < 0.001).

The optimal threshold value for the nomogram score determined by the NRI is 105. Subsequently, the patients were divided into two subgroups. Based on this cutoff value, 120 patients with a score less than or equal to 105 were classified as low risk recurrence group, while the remaining patients with a score greater than 105 were classified as high risk recurrence group (n = 13). According to pathological grading, the recurrence rates were 8% for low grade (n = 96) and 32% for high grade (n = 37), respectively. Based on the novel risk stratification system, the recurrence rate in the patients with scores of 0–105 (n = 120), 116–160 (n = 13) were 9% and 69%, respectively (**Table 2**). The predicted probabilities of recurrence-free survival were plotted as a Kaplan-Meier curve. Kaplan-Meier curve also consistently showed significant differences in postoperative recurrence between the two groups (P < 0.001) (**Figure 6**). The incidence of recurrence of the high risk cohort was significantly higher than that of the low risk cohort (P < 0.001).

**Table 2:** Postoperative recurrence in each group of the novel algorithm and WHO grade.

Prediction method	Group	Score	Postoperative recurrence rate (%)
Novel algorithm	Low risk	0-115	9
WHO grade	Low grade	—	8
Novel algorithm	High risk	116-160	69
WHO grade	High grade	—	32

## Discussion

We developed and validated a nomogram by using the Lasso-logistic model for postoperative recurrence prediction of meningioma in this study. To our knowledge, this is the first time that this novel algorithm is used to predict postoperative recurrence of meningioma. In this study, we also first discovered that the nomogram to predict the risk of meningioma recurrence based on pathological grade, conventional MR (enhanced tumor heterogeneity), and functional MR (rADC) was superior to WHO histopathological grade alone. The AUC value and the calibration curve also demonstrated that our nomogram had a good predictive performance. This result was further supported by the DCA and NRI. As a complement to pathological grade, non-invasive image-based techniques will help to better determine the risk of tumor recurrence and develop individualized treatment strategies (3, 6).

In univariate analysis, we found that pathological grade, tumor shape, peritumoral edema volume, peritumoral edema index, tumor volume, heterogeneous tumor enhancement, cystic change or necrosis, venous sinus invasion, arachnoid layer, age, and rADC were significantly associated with prognosis. Previous studies have investigated the relationship between potential factors and recurrence risk. Cohen-Inbar et al. have shown that older patients undergoing resection of meningiomas have higher recurrence rates compared with younger patients (14). Likewise, this study suggested that age proved to inversely correlate with prognosis. The observed association between cystic degeneration and high risk group was consistent with prior studies (8). Cyst formation in meningiomas may be associated with the rapid growth or aggressivity of tumor cells, including cystic variation, ischemic necrosis, direct liquid secretion by tumor cells, and absorption of intratumoral haemorrhage (4, 8, 12, 15). Our results indicated significant differences in tumor shape and volume between the recurrent and non-recurrent groups. Similarly, several researchers found that irregular tumor shape and larger tumor volumes were associated with greater proliferative potential, and active proliferation leads to increased recurrence after resection (4, 6, 15–17). In addition, we demonstrated that peritumoral brain edema (PTBE), including peritumoral edema volume and peritumoral edema index, was strongly associated with prognosis. PTBE was considered to predict brain invasion in most series (18, 19). Leehi et al. revealed that radiomics combined with peritumoral edema and interface showed high performance for the prediction of brain invasion based on a large cohort (n = 641) in both training (AUC: 0.97, 95% CI: 0.95–0.98) and independent validation sets (AUC: 0.91, 95% CI: 0.84–0.98) (19). However, further study is needed to reveal the relation between PTBE and aggressive biological behaviour because there are several in-consistent reports (4, 13). We also observed that disruption of the arachnoid layer was in relation to increased risk of recurrence, which closely agreed with previous studies (11, 15). The presence of the arachnoid layer indicates a slow and suppressing growth manner of the tumor (15). However, the loss of integrity of the arachnoid layer is not characteristic of brain invasion on microscopic analyses (20). Thus, the correlations between arachnoid layer and prognosis remain controversial. In the attainable data, venous sinus invasion was difficult to include because it is difficult to clearly distinguish the intravenous sinus tumor by contrast-enhanced MRI alone. Hence, we assessed venous sinus invasion by combining radiological data and surgical record judgment. We noted that venous sinus invasion was remarkably related to a higher recurrence rate, highlighting the importance of evaluating venous sinus involvement by adjacent lesions. Kei et al. reported that it was difficult to completely remove the entire tumor in a patient with an extensive venous sinus infiltrating tumor (21). Nevertheless, several studies recently reported that the recurrence rate in patients with venous sinus infiltration was rather variable, and there was no significant difference in incidence between patients with and without complete sinus resection (22–24). These findings suggest that postoperative recurrence of venous sinus invasion cannot simply be attributed to the residual tumor. Notably, there was a significant association between high grade tumors and venous sinus invasion by chi-square tests (P = 0.043) in this study, which suggested that tumor involving venous sinuses tended to be more aggressive.

Heterogeneous tumor enhancement is thought to be due to the uneven distribution of dividing cells and tumor necrosis, which may indicate the presence of local necrosis and a higher degree of malignancy in meningiomas (6, 11, 15, 20, 25). Some studies have shown that small focal necrosis in meningioma is related to a higher recurrence rate (11, 26, 27). Necrosis is presumed to result from hypoxia due to cellular undernutrition and hypermetabolism, suggesting that it may be associated with more aggressive progression (27). Hypoxic tumor cells in necrotic areas may dedifferentiate and develop into malignant cells (27). Inflammatory cells tend to cluster in necrotic areas, while degraded tumor cells release proinflammatory cytokines which may stimulate angiogenesis and tumor progression (28). Most studies have suggested that heterogeneous enhancement is an independent predictor of high grade meningiomas and postoperative recurrence (4, 6, 11, 12, 14, 15, 20, 25). Durand et al. showed the presence of heterogeneous contrast seemed to predict aggressive behavior in high grade meningiomas (25). Our study is consistent with previous studies confirming that heterogeneous tumor enhancement was an independent predictor of meningioma recurrence.

Diffusion weighted imaging and associated ADC maps are widely used in oncology to differentiate between malignant and non-malignant lesions. ADC is generally lower in malignant lesions than in benign lesions and surrounding normal tissues. The non-invasive observation of the distribution of displacements driven by water diffusion provides unique clues to the fine structural characteristics and geometrical structure of tissues and how these characteristics change with physiological or pathological states (6, 13, 29–31). William et al. have shown that meningiomas with non-Simpson grade I resection and low ADC have a significantly increased risk of progression/recurrence (P/R) (6). Ching‐Chung et al. have demonstrated that lower ADC values (< 0.83 × 10^− 3^ mm^2^/s)/ratios (<1.09) were significantly associated with P/R. Moreover, ROC analysis indicated that ADC ratio (AUC = 0.91; Sensitivity = 0.79; Specificity = 0.94) had a better performance for differentiating skull base meningiomas with and without P/R than ADC value (AUC = 0.86; Sensitivity = 0.73; Specificity = 0.88) (13). Regrettably, the above study did not further include rADC in the multivariate analysis. As far as we know, this is the first study to comprehensively discuss the relationship between rADC and prognosis in meningiomas. Our study was the first to confirm that rADC was a strong predictor of postoperative recurrence of meningioma. Yuan et al. observed that the ADC showed a moderate negative correlation with the Ki67 proliferation index in murine models of rhabdomyosarcoma (r = − 0.543, P = 0.003), which was generally consistent with what we observed in 66 meningioma patients (r = -0.305, P = 0.023) (31). Moreover, Manabu et al. found a stronger correlation between rADC and cell density than ADC (30). This may be because rADC to some extent eliminated the differences between the individuals and the scanning instruments. Therefore, rADC can more accurately reflect the proliferative activity and microstructure of the tumor.

**Figure 7:**
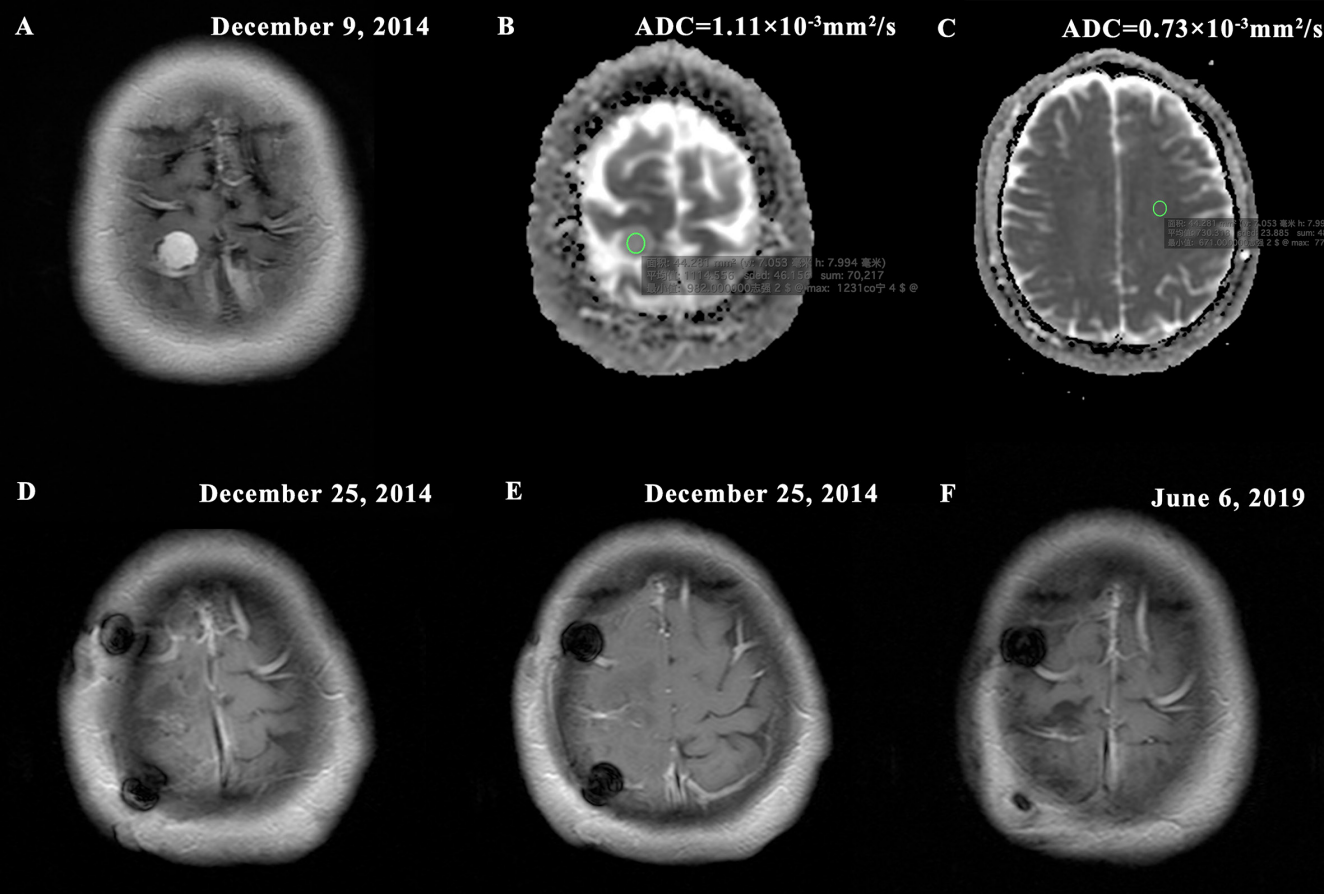
Typical MR images of a WHO II grade meningioma with non-invasive biological behavior demonstrating **(A)** homogeneous enhancement (presurgical T1-postcontrast); **(B)** high tumor apparent diffusion coefficient (ADC) = 1.11×10-3mm2/s (ADC map); **(C)** contralateral normal appearing white matter (NAWM) ADC = 0.73×10-3mm2/s and rADC = 1.52; **(D–E)** Simpson grade I resection (postsurgical T1-postcontrast); **(F)** no imaging manifestation of tumor recurrence (postoperative T1-postcontrast). The new risk stratification puts the patient in the low risk group for recurrence (nomogram score = 50). The patient was followed up for 55 months without tumor recurrence.

At present, several studies still suggested that the histological grade remains the most important indicator of postoperative recurrence of meningioma (2, 11, 32). In this study, we also demonstrated a significant correlation between pathological grade and postoperative recurrence in patients who underwent total resection (P < 0.001). Although grade II/III meningiomas have more aggressive biological behavior and might exhibit faster recurrence, there are also some high grade meningiomas that biologically behave more like benign lesions by our observation (**Figure 7**). Likewise, many studies showed that it was inadequate to predict postoperative recurrence and develop individualized treatment strategies based solely on WHO classification (3, 6, 8, 9, 11–13, 17). Consequently, in this study, we established a prediction model integrating clinical data, imaging, and pathology for tumor prognosis, and explored and developed personalized treatment strategies base on this model.

On the other hand, sex, preoperative SKP, T2 relative high signal, dural tail sign, bone change, calcification, tumor location, and postoperative adjuvant radiotherapy were not independent risk factors in our study. There were controversial reports about the influence of sex on meningioma recurrence. Some studies revealed that male patients had a high risk of recurrence (11, 12, 33), while others did not (6, 9, 13, 17, 25). Although we observed a trend towards an association between preoperative SKP and recurrence, it did not reach significance. We agreed with previous studies that the T2 relative high signal was generally not correlated with prognosis (4, 6, 12, 13, 15). The presence of linear enhancement of the dural caudate or adjacent dura may distinguish the meningioma from other intracranial masses following the use of contrast agents (34). Although the dural tail sign is helpful in the diagnosis of meningioma, it has little prognostic significance (4, 6, 11, 13). Anthofer et al. found that bone invasion was an important risk factor in meningiomas adjacent to major venous sinuses. In contrast, there was no statistical difference between bone invasion and recurrence in our study. The presence of tumor calcification may have lower proliferative potential than noncalcified tumor, but not an increased risk of recurrence (4, 6). McGovern et al. have suggested that advances in surgical techniques have improved the grade of resection of skull base tumors in the modern case series (35). Skull base meningiomas were no longer a risk factor for postoperative recurrence. Clinically, adjuvant radiotherapy (RT) is a standard procedure in many institutions after subtotal resection (STR) of high grade meningiomas, but the role of RT as adjuvant therapy after gross total resection (GTR) is still unclear (2, 6, 36). In fact, we found no apparent effect of radiotherapy on the recurrence of high-grade meningiomas (with radiotherapy *vs*. without radiotherapy: 31% *vs*. 33%), which may indicate that WHO classification is not an ideal indicator of radiotherapy. On the contrary, in the high-risk population screened by our model, the recurrence rate of patients with and without radiotherapy was 43% and 100%, respectively. Notably, all 4 high-risk patients without recurrence received radiotherapy. Although further studies are needed to evaluate this finding, it indicates that our model may be a potential method to screen patients for postoperative radiotherapy.

In a previous study, William et al. reviewed 144 patients with a pathologically confirmed meningioma and selected Simpson grade and ADC value to constructed multivariate Cox proportional hazards models according to maximizing predictive ability and minimizing model complexity (HC, 0.73; AIC, 244; BIC, 250). On univariate Cox proportional hazards methods, there was only a marginal correlation between histopathological grade and prognosis (HR, 2.01; 95% CI, 0.929-0.4.33; P = 0.076). Thus, William et al. suggested that the combination of preoperative ADC and extent of surgical resection was superior to histopathological grading in predicting the hazard of tumor recurrence (6). This is undoubtedly an exciting finding, but we cannot take this conclusion with complete confidence because it is not convincing that pathological grade does not make a statistically significant difference in predicting postoperative recurrence of meningioma. Therefore, we tried to use conventional MRI, functional MRI (fMRI), and clinical data as a complement to the pathological grade and developed a predictor based on a novel algorithm. The result of the ROC curve proved that the model by integrating pathological grade, heterogeneous tumor enhancement and rADC (AUC = 0.853, 95%CI: 0.764-0.942) had a higher prognostic value than the pathological grade alone (AUC = 0.689, 95%CI: 0.573-0.806), which was further proved by DCA and NRI. Zhu et al. used multiple logistic regression analysis to establish a scoring system that accurately predicted recurrence in combination with conventional MRI and pathological grading, and divided patients into the following 4 subgroups. The incidences of recurrence in each subgroup were as follows: subgroup 1 (1.2%); subgroup 2 (5.7%); subgroup 3 (26.1%); subgroup 4 (66.7%) (P < 0.001) (11). However, consistency between predicted probability and actual probability was not assessed by using calibration curves in their study. Besides, the repeatability and reproducibility of the model were not tested by performing validation of the prediction model. In contrast, our study made up for the above deficiencies. While conventional MRI has limited ability to predict recurrence of meningiomas after radical resection. ADC map renders microstructure regard to cellular density and tumor matrix (29). Therefore, in addition to conventional MRI, our work also included fMRI to improve the predictive potential of meningiomas after treatment. Recently, Farshad et al. generated and validate predictors of postoperative meningioma recurrence and a nomogram based on the Cox model that incorporated methylation, pathological grade, and Simpson grade recurrence in a multicenter retrospective study (3). The discrimination of the meningioma recurrence was up to 82% in combined validation cohorts (AUC = 0.82, 95% CI: 0.76–0.87) (3). In this work, we used Lasso to select variables, which not only improved the accuracy of prediction, but also ensured the interpretability and stability of the model, and reduced the complexity of the model (37). In brief, this method can well overcome the shortcomings of traditional methods. Although the Lasso-logistic model, which combines pathological grading and imaging features, performed similarly to the methylated group-based model, non-invasive imaging is more convenient, economical, reproducible, and has greater potential for continuous monitoring.

Although our work can improve the accuracy of prediction of postoperative meningioma recurrence, this study still has some limitations. First of all, our study is a retrospective study at a single institution. Therefore, it lacks external validation to test the generalization ability of the model. Furthermore, the sample size was not large enough, which may lead to statistical bias. Also, follow-up time might be still not long enough for benign tumors. In the future, prospective studies based on large-scale multiple centres are needed to verify the reliability and generalization of our model. Finally, the expensive examination fee of MRI may also limit our research objectively.

In this study, we spotted the independent prognostic factor for post-treatment recurrence of meningioma, and combined conventional magnetic resonance with functional magnetic resonance using the Lasso-logistic algorithm to establish a simple and reliable meningioma prediction model. We found that non-invasive imaging techniques were used to supplement the pathological grade, which had a higher prognostic value than the pathologic grade alone and helped to distinguish between patients at different risks of recurrence. Moreover, the proposed model can help in optimizing the individualized design of clinical therapy.

## Data Availability Statement

The raw data supporting the conclusions of this article will be made available by the authors, without undue reservation.

## Ethics Statement

The studies involving human participants were reviewed and approved by the Institutional Review Board (Fujian Medical University Union Hospital). Written informed consent from the participants’ legal guardian/next of kin was not required to participate in this study in accordance with the national legislation and the institutional requirements. Written informed consent was not obtained from the individual(s), nor the minor(s)’ legal guardian/next of kin, for the publication of any potentially identifiable images or data included in this article.

## Author Contributions

Conceptualization, YX, LL, and RZ. Data processing: RZ, LL, and YS. Data collection: RZ and XC. Methodology: YX, LL, RZ, and XC. Statistical analysis: RZ and XC. Software: RZ and JC. Manuscript drafting: RZ and XC. Manuscript proofreading: JC and PJ. Critical revision: YX and LL. Manuscript approval: all authors. All authors contributed to the article and approved the submitted version.

## Funding

Sponsored by Joint Funds for the Innovation of Science and Technology, Fujian province (Grant number: 2018Y9044); Fujian Provincial Health Technology Project (Grant number: 2020GGA039); Startup Fund for Scientific Research, Fujian Medical University (Grant number: 2019QH1062); Innovation and Entrepreneurship Training Program of Fujian Medical University (Grant number: S202110392011).

## Conflict of Interest

Author YS was employed by Siemens, Healthineers Ltd.

The remaining authors declare that the research was conducted in the absence of any commercial or financial relationships that could be construed as a potential conflict of interest.

## Publisher’s Note

All claims expressed in this article are solely those of the authors and do not necessarily represent those of their affiliated organizations, or those of the publisher, the editors and the reviewers. Any product that may be evaluated in this article, or claim that may be made by its manufacturer, is not guaranteed or endorsed by the publisher.

## References

[1] Ostrom Q Patil N Cioffi G Waite K Kruchko C Barnholtz-Sloan J. CBTRUS Statistical Report: Primary Brain and Other Central Nervous System Tumors Diagnosed in the United States in 2013-2017. Neuro Oncol (2020) 22:iv1–iv96. doi: 10.1093/neuonc/noaa200.s 33123732 PMC7596247

[2] Goldbrunner R Minniti G Preusser M Jenkinson M Sallabanda K Houdart E. EANO Guidelines for the Diagnosis and Treatment of Meningiomas. Lancet Oncol (2016) 17(9):e383–91. doi: 10.1016/s1470-2045(16)30321-7 27599143

[3] Nassiri F Mamatjan Y Suppiah S Badhiwala J Mansouri S Karimi S. DNA Methylation Profiling to Predict Recurrence Risk in Meningioma: Development and Validation of a Nomogram to Optimize Clinical Management. Neuro Oncol (2019) 21(7):901–10. doi: 10.1093/neuonc/noz061 PMC6620635 31158293

[4] Spille D Sporns P Heß K Stummer W Brokinkel B. Prediction of High-Grade Histology and Recurrence in Meningiomas Using Routine Preoperative Magnetic Resonance Imaging: A Systematic Review. World Neurosurg (2019) 128:174–81. doi: 10.1016/j.wneu.2019.05.017 31082555

[5] Ildan F Erman T Göçer A Tuna M Bağdatoğlu H Cetinalp E. Predicting the Probability of Meningioma Recurrence in the Preoperative and Early Postoperative Period: A Multivariate Analysis in the Midterm Follow-Up. Skull Base (2007) 17(3):157–71. doi: 10.1055/s-2007-970554 PMC1888737 17973029

[6] Hwang W Marciscano A Niemierko A Kim D Stemmer-Rachamimov A Curry W. Imaging and Extent of Surgical Resection Predict Risk of Meningioma Recurrence Better Than WHO Histopathological Grade. Neuro Oncol (2016) 18(6):863–72. doi: 10.1093/neuonc/nov285 PMC4864259 26597949

[7] Weber D Ares C Villa S Peerdeman S Renard L Baumert B. Adjuvant Postoperative High-Dose Radiotherapy for Atypical and Malignant Meningioma: A Phase-II Parallel non-Randomized and Observation Study (EORTC 22042-26042). Radiother Oncol (2018) 128(2):260–5. doi: 10.1016/j.radonc.2018.06.018 29960684

[8] Kalasauskas D Kronfeld A Renovanz M Kurz E Leukel P Krenzlin H. Identification of High-Risk Atypical Meningiomas According to Semantic and Radiomic Features. Cancers (2020) 12(10):2942. doi: 10.3390/cancers12102942 PMC7599676 33053798

[9] Liu Y Chotai S Chen M Jin S Qi S Pan J. Preoperative Radiologic Classification of Convexity Meningioma to Predict the Survival and Aggressive Meningioma Behavior. PloS One (2015) 10(3):e0118908. doi: 10.1371/journal.pone.0118908 25786236 PMC4364713

[10] Louis D Perry A Reifenberger G von Deimling A Figarella-Branger D Cavenee W. The 2016 World Health Organization Classification of Tumors of the Central Nervous System: A Summary. Acta Neuropathol (2016) 131(6):803–20. doi: 10.1007/s00401-016-1545-1 27157931

[11] Zhu Z Wang C Xu J Wang C Xia L Li Q. A Quantified Risk-Scoring System for the Recurrence of Meningiomas: Results From a Retrospective Study of 392 Patients. Front Oncol (2020) 10:585313. doi: 10.3389/fonc.2020.585313 33123487 PMC7570434

[12] Spille D Adeli A Sporns P Heß K Streckert E Brokinkel C. Predicting the Risk of Postoperative Recurrence and High-Grade Histology in Patients With Intracranial Meningiomas Using Routine Preoperative MRI. Neurosurg Rev (2021) 44(2):1109–17. doi: 10.1007/s10143-020-01301-7 PMC8450214 32328854

[13] Ko C Lim S Chen T Chen J Li C Shiue Y. Prediction of Progression in Skull Base Meningiomas: Additional Benefits of Apparent Diffusion Coefficient Value. J Neurooncol (2018) 138(1):63–71. doi: 10.1007/s11060-018-2769-9 29353434

[14] Cohen-Inbar O Soustiel JF Zaaroor M. Meningiomas in the Elderly, the Surgical Benefit and a New Scoring System. Acta Neurochir (2010) 152(1):87–97. doi: 10.1007/s00701-009-0552-6 19936609

[15] Li H Zhao M Jiao Y Ge P Li Z Ma J. Prediction of High-Grade Pediatric Meningiomas: Magnetic Resonance Imaging Features Based on T1-Weighted, T2-Weighted, and Contrast-Enhanced T1-Weighted Images. World Neurosurg (2016) 91:89–95. doi: 10.1016/j.wneu.2016.03.079 27046015

[16] Ho D Hsu C Ting L Chiang H. Histopathology and MIB-1 Labeling Index Predicted Recurrence of Meningiomas: A Proposal of Diagnostic Criteria for Patients With Atypical Meningioma. Cancer (2002) 94(5):1538–47. doi: 10.1002/cncr.10351 11920512

[17] Fujimoto T Ishida Y Uchiyama Y Nakase H Sakaki T Nakamura M. Radiological Predictive Factors for Regrowth of Residual Benign Meningiomas. Neurol Med Chir (2011) 51(6):415–22. doi: 10.2176/nmc.51.415 21701104

[18] Hess K Spille D Adeli A Sporns P Brokinkel C Grauer O. Brain Invasion and the Risk of Seizures in Patients With Meningioma. J Neurosurg (2018) 130(3):789–96. doi: 10.3171/2017.11.Jns172265 29701550

[19] Joo L Park J Park S Nam S Kim Y Kim J. Extensive Peritumoral Edema and Brain-to-Tumor Interface MRI Features Enable Prediction of Brain Invasion in Meningioma: Development and Validation. Neuro Oncol (2021) 23(2):324–33. doi: 10.1093/neuonc/noaa190 PMC8631067 32789495

[20] Adeli A Hess K Mawrin C Streckert E Stummer W Paulus W. Prediction of Brain Invasion in Patients With Meningiomas Using Preoperative Magnetic Resonance Imaging. Oncotarget (2018) 9(89):35974–82. doi: 10.18632/oncotarget.26313 PMC6267603 30542511

[21] Yamashiro K Hasegawa M Higashiguchi S Kato H Hirose Y. Intravenous Sinus Meningioma With Intraluminal Extension to the Internal Jugular Vein: Case Report and Review of the Literature. Br J Neurosurg (2020), 1–6. doi: 10.1080/02688697.2020.1777258 32536219

[22] Sughrue M Rutkowski M Shangari G Parsa A Berger M McDermott M. Results With Judicious Modern Neurosurgical Management of Parasagittal and Falcine Meningiomas. Clinical article. J Neurosurg (2011) 114(3):731–7. doi: 10.3171/2010.9.Jns10646 20950085

[23] Anthofer J Seidel-Schulz R Proescholdt M Brawanski A Schebesch K. Meningiomas Adjacent to Major Venous Sinuses-Clinical Outcome and Recurrence. World Neurosurg (2017) 104:560–6. doi: 10.1016/j.wneu.2017.05.025 28512040

[24] Maiuri F Donzelli R Pagano S Mariniello G. The Management of the Venous Sinuses During Surgery for Posterior Fossa Meningiomas. World Neurosurg (2019) 125:357–63. doi: 10.1016/j.wneu.2019.02.032 30797929

[25] Durand A Labrousse F Jouvet A Bauchet L Kalamaridès M Menei P. WHO Grade II and III Meningiomas: A Study of Prognostic Factors. J Neurooncol (2009) 95(3):367–75. doi: 10.1007/s11060-009-9934-0 19562258

[26] McLean C Jolley D Cukier E Giles G Gonzales M. Atypical and Malignant Meningiomas: Importance of Micronecrosis as a Prognostic Indicator. Histopathology (1993) 23(4):349–53. doi: 10.1111/j.1365-2559.1993.tb01218.x 8300070

[27] Góes P Santos B Suzuki F Salles D Stávale J Cavalheiro S. Necrosis Is a Consistent Factor to Recurrence of Meningiomas: Should It be a Stand-Alone Grading Criterion for Grade II Meningioma? J Neurooncol (2018) 137(2):331–6. doi: 10.1007/s11060-017-2721-4 29270884

[28] Vakkila J Lotze MT. Inflammation and Necrosis Promote Tumour Growth. Natrevimmunol (2004) 4(8):641–8. doi: 10.1038/nri1415 15286730

[29] Le Bihan D. Apparent Diffusion Coefficient and Beyond: What Diffusion MR Imaging can Tell Us About Tissue Structure. Radiology (2013) 268(2):318–22. doi: 10.1148/radiol.13130420 23882093

[30] Kinoshita M Arita H Okita Y Kagawa N Kishima H Hashimoto N. Comparison of Diffusion Tensor Imaging and C-Methionine Positron Emission Tomography for Reliable Prediction of Tumor Cell Density in Gliomas. J Neurosurg (2016) 125(5):1136–42. doi: 10.3171/2015.11.Jns151848 26918477

[31] Yuan Y Zeng D Liu Y Tao J Zhang Y Yang J. DWI and IVIM Are Predictors of Ki67 Proliferation Index: Direct Comparison of MRI Images and Pathological Slices in a Murine Model of Rhabdomyosarcoma. Eur Radiol (2020) 30(3):1334–41. doi: 10.1007/s00330-019-06509-w 31705255

[32] Marciscano A Stemmer-Rachamimov A Niemierko A Larvie M Curry W Barker F. Benign Meningiomas (WHO Grade I) With Atypical Histological Features: Correlation of Histopathological Features With Clinical Outcomes. J Neurosurg (2016) 124(1):106–14. doi: 10.3171/2015.1.Jns142228 26274991

[33] Escribano Mesa J Alonso Morillejo E Parrón Carreño T Huete Allut A Narro Donate J Méndez Román P. Risk of Recurrence in Operated Parasagittal Meningiomas: A Logistic Binary Regression Model. World Neurosurg (2018) 110:e112–8. doi: 10.1016/j.wneu.2017.10.087 29107168

[34] Alorainy I. Dural Tail Sign in Spinal Meningiomas. Eur J Radiol (2006) 60(3):387–91. doi: 10.1016/j.ejrad.2006.06.012 16876365

[35] McGovern S Aldape K Munsell M Mahajan A DeMonte F Woo S. A Comparison of World Health Organization Tumor Grades at Recurrence in Patients With non-Skull Base and Skull Base Meningiomas. J Neurosurg (2010) 112(5):925–33. doi: 10.3171/2009.9.Jns09617 19799498

[36] Delgado-López P Corrales-García E. Role of Adjuvant Radiotherapy in Atypical (WHO Grade II) and Anaplastic (WHO Grade III) Meningiomas: A Systematic Review. Clin Transl Oncol (2021) 23(2):205–21. doi: 10.1007/s12094-020-02434-3 32651886

[37] Tibshirani RJ. Regression Shrinkage and Selection *via* the LASSO. J R Stat Soc Ser B Methodol (1996) 73(1):273–82. doi: 10.1111/j.2517-6161.1996.tb02080.x

