# Supplementary material for: A Novel MRI-Based Risk Stratification Algorithm for Predicting Postoperative Recurrence of Meningioma: More Benefits to Patients
Source: Front Oncol. 2021 Oct 19;11:737520. doi: 10.3389/fonc.2021.737520 (PMC8560899; doi:10.3389/fonc.2021.737520)
Supplement: Supplementary file 1 [file DataSheet_1.docx]

Supplementary Appendix

**E1:** **MRI Protocols**

The protocols of 1.5T (Siemens, MAGNETOM Avanto) MR scanner were as the following: axial T1-weighted spin-echo (T1WI) (TR/TE, 1990/9 ms; FOV, 22 cm; slice thickness/spacing, 5 mm/1.5 mm; matrix, 320 × 228; voxel size, 0.7 × 0.7 × 5 mm^3^), T2-weighted imaging (T2WI) (fast spin-echo) (5360/109 ms; FOV, 22 cm; slice thickness/spacing, 5 mm/1.5 mm; matrix, 320 × 228; voxel size, 0.6 × 0.6 × 5 mm^3^), fluid attenuated inversion recovery (FLAIR) (TR/TE, 9000/95 ms; FOV, 22 cm; slice thickness/spacing, 5 mm/1.5 mm; matrix, 256 × 186; voxel size, 0.9 × 0.9 × 5 mm^3^ ), and T2-weighted gradient-recalled echo (GRE) (TR/TE, 830/25 ms; FOV, 22 cm; slice thickness/spacing, 5 mm/1.5 mm; matrix, 256 × 186; voxel size, 0.8 × 1.0 × 5 mm^3^). Contrast-enhanced images obtained in axial and coronal and sagittal T1WI with fat saturation (1950/10 ms; FOV, 23 cm; slice thickness/spacing, 5 mm/1.5 mm; matrix, 256 × 256, voxel size, 0.9 × 0.9 × 5 mm^3^) were performed after intravenous administration of 0.1 mmol/kg of body weight of gadobutrol (Gadovist, Bayer Healthcare, Germany). The DWI was performed by applying sequentially in the x, y, and z directions with the following parameters: TR/TE, 3800/100 ms; FOV, 23 cm; flip angle, 90 degrees; slice thickness/spacing, 5 mm/1.5 mm; matrix 150 × 150; voxel size, 1.6 × 1.6 × 5 mm^3^; b = 0, 500, and 1000 sec/mm^2^. ADC maps were obtained from these imaging data.

The protocols of 3.0T (Siemens, MAGNETOM Trio) MR scanner were as following: axial T1WI (TR/TE, 2000/25 ms; FOV, 20 cm; slice thickness/spacing, 5 mm/1.5 mm; matrix, 320 × 240; voxel size, 0.7 × 0.9 × 5 mm^3^), T2WI (TR/TE, 6000/91 ms; FOV, 20 cm; slice thickness/spacing, 5 mm/1.5 mm; matrix, 320 × 240; voxel size, 0.7 × 0.7 × 5 mm^3^), FLAIR (9000/94 ms; FOV, 20 cm; slice thickness/spacing, 5 mm/1.5 mm; matrix, 256 × 186; voxel size, 0.9 × 0.9 × 5 mm^3^), and T2-weighted GRE (TR/TE, 830/25 ms; FOV, 20 cm; slice thickness/spacing, 5 mm/1.5 mm; matrix, 288 × 186; voxel size, 0.8 × 1.0 × 5 mm^3^). Contrast-enhanced axial and coronal and sagittal T1WI with fat saturation (TR/TE, 2500/25 ms; FOV, 20 cm; slice thickness/spacing, 5 mm/1.5 mm; matrix, 256 × 256; voxel size, 0.9 × 0.9 × 5 mm^3^) with intravenous administration of 0.1 mmol/kg of gadobutrol (Gadovist, Bayer Healthcare, Germany). The DWI was performed by applying sequentially in the x, y, and z directions with the following parameters: TR/TE, 3100/91 ms; FOV, 20 cm; flip angle, 90 degrees; slice thickness/spacing, 5 mm/1.5 mm; matrix 192 × 192; voxel size, 1.2 × 1.2 × 5 mm^3^; b = 0, 500, and 1000 sec/mm2. ADC maps were obtained from these imaging data.

The protocols of 3.0T (GE Healthcare, Discovery MR750) MR scanner were as following: axial T1WI (TR/TE, 3400/24 ms; FOV, 22 cm; slice thickness/spacing, 5 mm/1.5 mm; matrix, 320 × 256; voxel size, 0.7 × 0.9 × 5 mm^3^), T2WI (TR/TE, 6400/140 ms; FOV, 22 cm; slice thickness/spacing, 5 mm/1.5 mm; matrix, 384 × 320; voxel size, 0.6 × 0.6 × 5 mm^3^), FLAIR (TR/TE, 9000/95 ms; FOV, 22 cm; slice thickness/spacing, 5 mm/1.5 mm; matrix, 288 × 224; voxel size, 0.8 × 1.0 × 5 mm^3^), and T2-weighted GRE (TR/TE, 550/15 ms; FOV, 22 cm; slice thickness/ spacing, 5 mm/1.5 mm; matrix, 320 × 224; voxel size, 0.8 × 1.1 × 5 mm^3^). Contrast-enhanced axial and coronal and sagittal T1WI with fat saturation (TR/TE, 1800/22 ms; FOV, 22 cm; slice thickness/spacing, 5 mm/1.5 mm; matrix, 320 × 256; voxel size, 0.7 × 0.9 × 5 mm^3^) after intravenous administration of 0.1 mmol/kg of gadobenate Dimeglumine (Multihance, Bracco, Italy). The DWI was performed by applying sequentially in the x, y, and z directions with the following parameters: TR/TE, 8000/64 ms; FOV, 22 cm; flip angle, 90 degrees, slice thickness/spacing, 5 mm/1.5 mm; matrix 192 × 192; voxel size, 1.8 × 1.8 × 5 mm^3^; b = 0, 500, and 1000 sec/mm2. ADC maps were obtained from these imaging data.

# E2: The formula for Lasso regression

The lasso estimate is defined by

$\begin{matrix} \left( \hat{\alpha},\hat{\beta} \right)=\arg\min\left\{ {\sum_{i=1}^{N} \left( y_{i}-\alpha-\sum_{j} \beta_{j}x_{ij} \right)}^{2} \right\} & subject to \sum_{j} \left| \beta_{j} \right|\leq t. \end{matrix}$

Least Absolute Shrinkage and Selection Operator is a new variable selection method proposed by Robert Tibshirani (1996) inspired by ridge regression and "Nonnegative Garrote" method. The Lasso method uses the absolute value function of the model coefficients as a penalty to compress the model coefficients, making some regression coefficients smaller, and even making the coefficients with smaller absolute values automatically compress to 0. In the selection of variables, it also realizes the estimation of parameters. Variable selection of the main goals include forecasting accuracy and interpretability of the model and stability as well as the complexity of the model, the traditional model selection methods such as partial least squares method, stepwise regression, ridge regression, principal component analysis method can only achieve the purpose of the part, the effect of the Lasso method in terms of variable selection, superior to the above several ways, This method can overcome the shortcomings of traditional methods in model selection.

# Supplementary Table

| **The 2016 WHO classification of meningiomas** |
| --- |
| **WHO Grade I**: < 4 mitoses/10 HPF |
| Meningothelial meningioma |
| Fibrous (fibroblastic) meningioma |
| Transitional (mixed) meningioma |
| Psammomatous meningioma |
| Angiomatous meningioma |
| Microcystic meningioma |
| Secretory meningioma |
| Lymphoplasmacyte-rich meningioma |
| Metaplastic meningioma |
| **WHO Grade II:** |
| Atypical meningioma (4–19 mitoses/10 HPF or brain Invasion) |
| Clear cell meningioma |
| Chordoid meningioma |
| **WHO Grade III:** |
| Anaplastic (malignant) meningioma ≥ 20 mitoses/10 HPF |
| Rhabdoid meningioma |
| Papillary meningioma |

Note: HPF indicate high power field

#

# Supplementary Figure


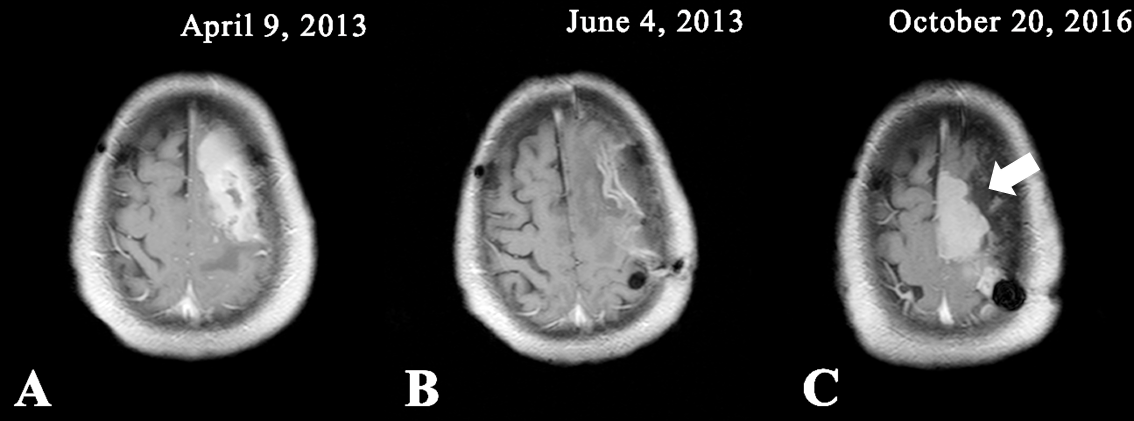


**A WHO I grade meningioma was pathologically confirmed as postoperative recurrence.** (A) Preoperative MR enhanced T1-weighted image showing a mass in left cerebral convexity. (B) Postoperative MR enhanced T1-weighted image confirmed Simpson grade I resection. (C) Follow-up MR enhanced T1-weighted images showing a new contrast-enhanced mass in the previous resection cavity (arrow).
